# The Protective Effects of Perceived Control During Repeated Exposure to Aversive Stimuli

**DOI:** 10.3389/fnins.2021.625816

**Published:** 2021-02-03

**Authors:** Kainan S. Wang, Mauricio R. Delgado

**Affiliations:** ^1^McLean Imaging Center, McLean Hospital, Belmont, MA, United States; ^2^Harvard Medical School, Boston, MA, United States; ^3^Department of Psychology, Rutgers University, Newark, NJ, United States

**Keywords:** perceived control, ventromedial prefrontal cortex, learned helplessness, passivity, avoidance behavior

## Abstract

The ability to perceive and exercise control is a major contributor to our mental and physical wellbeing. When faced with uncontrollable aversive stimuli, organisms develop heightened anxiety and become unwilling to exert effort to avoid the stimuli. In contrast, when faced with controllable aversive stimuli, organisms demonstrate behavioral vigor via avoidance attempts toward trying to seek and exercise control over the environment. As such, controllability confers protective effects against reduced avoidance motivation trigged by aversive environments. These observations beg the question of whether controllability can be potent enough to reverse passivity following repeated exposure to uncontrollable aversive stimuli and how this protective effect is encoded neurally. Human participants performed a Control in Aversive Domain (CAD) task where they were first subjected to a series of repeated uncontrollable aversive stimuli (i.e., aversive tones) across several contexts that were followed by a series of controllable aversive stimuli in a novel context. Faced with persistent uncontrollability, participants significantly reduced their avoidance attempts over time and biased toward giving up. However, the subsequent presence of controllability rescued participants’ avoidance behavior. Strikingly, participants who responded more strongly to the protective effects of control also had greater ventromedial prefrontal cortical (vmPFC) activation—a region previously observed to be associated with encoding the subjective value of control. Taken together, these findings highlighted the protective effect conferred by perceived control against passivity and offered insights into the potential role of the vmPFC in controllable environments, with implications for understanding the beneficial influence of perceived control on adaptive behavior.

## Introduction

Our sense of control is governed by our perceived ability to influence the environment. This ability to perceive and exercise control—henceforth referred to as perceived control—serves an important role to help maintain and support a healthy psychological and physical state. As such, when an organism is faced with a situation where controllability is diminished or altogether absent, as represented by the dissociation of behavior and outcome, it is prone to develop passivity and heightened anxiety (Rodin, 1986; Wallston et al., 1987; Ryan and Deci, 2000). Passivity can translate into the action of giving up where the organism exhibits no overt avoidance or escape behaviors. This passivity and the associated reduction in avoidance motivation is often exacerbated in aversive environments, particularly when the organism is made to repeatedly endure uncontrollable aversive stimuli (for review see Maier and Seligman, 2016).

Prior work exploring the behavioral effects of perceived control have found that both animals and humans work harder and longer to obtain rewards or to avoid aversive outcomes when they perceive the environment to be controllable (Bongard, 1995; Bhanji et al., 2016; Wang et al., 2020). Moreover, when given the ability to do something about the external environment either in the form of performing an avoidance behavior (Hiroto, 1974) or granted the opportunity to exercise a choice (Rodin and Langer, 1977), human participants report stronger positive emotions and enhanced self-competence (Rodin, 1986; Deci and Ryan, 1987; Ly et al., 2019). In the same vein, having this perception of control is often also accompanied by stronger intrinsic motivation to learn to avoid or escape from aversive stimuli (Holmes and Jackson, 1975; Taub and Dollinger, 1975; Maier and Seligman, 1976; Quaglieri, 1980; Feather and Volkmer, 1988; Trusty and Macan, 1995). Given these findings, it is argued that perceived control over the environment has a protective effect on an organism’s avoidance motivation, particularly in an aversive environment that otherwise tends to induce passivity. When an organism believes that its behavior can reliably bring about desired outcomes, the organism—endowed with perceived control—is protected from feeling helpless in an aversive environment and maintains avoidance motivation. One open question that follows is whether the protective effect conferred by perceived control can reverse passivity associated with repeated exposure to uncontrollable aversive environment.

Here, we examined the protective effects of perceived control on human participants’ avoidance behavior and studied how such induced behavioral changes are subserved neurally. Previous research has largely implicated the ventromedial prefrontal cortex (vmPFC; Amat et al., 2005; Christianson et al., 2009) and the striatum (Leotti and Delgado, 2011, 2014) as critical regions associated with perceiving a sense of control. The vmPFC in particular, is important in encoding the subjective value of perceived control—how much people prefer to exert vs. give up control in order to attain a reward (Wang and Delgado, 2019). As such, we hypothesize that perceived control can not only engender avoidance behavior changes via its protective effects, it is likely that the vmPFC is involved in mediating such behavioral changes.

To test our hypotheses, we adapted a prior aversive behavioral paradigm using auditory tones (Hiroto, 1974) to design the Control in Aversive Domain (CAD) task. Briefly, the CAD task consisted of three different phases (i.e., *exposure, uncontrollable, and controllable*). The objective of the task was to first expose participants to uncontrollability (in the *exposure* phase), followed by testing for decreases in avoidance behavior (i.e., passivity) in a novel aversive but uncontrollable context (in the *uncontrollable* phase), and finally study the protective effects of controllability in rescuing avoidance behavior in a novel aversive context (in the *controllable* phase). To capture avoidance behavior and passivity, we asked participants to choose between an AVOID and a GIVE-UP option. Specifically, participants were asked to either make an active attempt to try and avoid the aversive stimulus (AVOID option) or make an active decision to give up trying to avoid the stimulus and accept it (GIVE-UP option). We predicted that the protective effects of controllability would be potent enough to rescue participants’ avoidance behavior and this behavioral change would be subserved by the vmPFC.

## Materials and Methods

### Participants

Thirty-one right-handed individuals (11 Males and 20 Females) between the ages of 18 and 37 [Mean (*M*) = 23.3, standard deviation (*SD*) = 5.1] were recruited from the Rutgers University community to perform two separate functional tasks: a Value of Control paradigm (published in Wang and Delgado, 2019) and the Control in Aversive Domain task (included here). Participants were prescreened for any history of psychiatric and neurological illness. They were given monetary compensation for their voluntary participation in the experiment. All participants provided written informed consent in accordance with the experimental protocol approved by the Rutgers University Institutional Review Board. Three participants did not complete the experiment due to equipment failure, while two participants were excluded from subsequent analyses due to complications (e.g., experienced phobia during the scanning session). Four participants were excluded based on the criteria that they had >50% missed trials in at least one experimental run. The final participant count was 22 (8 Males and 14 Females; M = 23.3, *SD* = 4.58), consistent with the desired sample size of 19, obtained from a power analysis for paired *t*-test conducted using G^∗^Power (version 3.1; Faul et al., 2007) according to the guidelines established by Cohen, 1992 (alpha = 0.05, power = 0.9, effect size = 0.8).

### Experimental Task and Design

We adapted a behavioral paradigm implemented in both animals (Maier and Seligman, 1976) and humans (Hiroto, 1974) to design the Control in Aversive Domain (CAD) neuroimaging task and tested the overarching hypothesis that vmPFC subserves the protective effects of controllability on avoidance behavior in an aversive context. This task was conceived with four factors in mind: (a) demonstrate behavioral differences in response to uncontrollable aversive and neutral contexts in a within-subject design; (b) repeated and uninterrupted exposure to uncontrollability across several contexts to examine its behavioral effect; (c) test for behavioral changes in response to controllability in a novel context *after* the exposure to repeatedly uncontrollability; (d) require participants to choose between AVOID or GIVE-UP options with a button press in order to balance motor- and effort- related confounds for neuroimaging analyses.

The CAD task comprised three sequential phases: *exposure, uncontrollable*, and *controllable.* In the *exposure* phase, we tested participants’ avoidance responses toward two cues (i.e., colored shapes) paired respectively with an aversive (i.e., 4,000 Hz) and a neutral (i.e., 500 Hz) tone. Both cues were presented in an uncontrollable context where participants had no real behavioral control and could never successfully avoid the tones. In the ensuing *uncontrollable* and *controllable* phases, we paired two novel cues respectively with a controllable and an uncontrollable aversive tone to investigate changes in participants’ avoidance behavior toward these novel contexts. Each phase will be described in more details in subsequent sections.

At the beginning of the experiment, participants were first asked to hear and rate the aversiveness [i.e., “How aversive (unpleasant) is the tone?”] of two tones on a Likert scale of 1–7. The aversive and neutral tones were designed to match in terms of their amplitude at 75 decibels (i.e., loudness), but differed in their frequency (i.e., pitch). The presentation of the tones was counterbalanced across participants.

#### Run Structure

**FIGURE 1:**
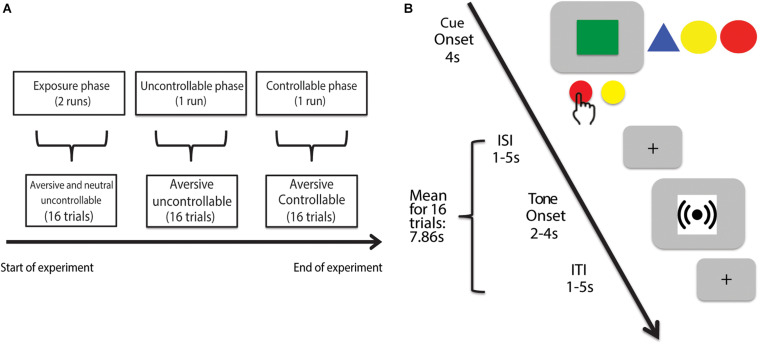
Experimental timeline of Control in Aversive Domain (CAD) task. **(A)** The CAD task consisted of three phases. The first phase comprised the *exposure* trials where participants responded to uncontrollable cues paired with either an aversive (4,000 Hz) or neutral (500 Hz) tone. The second phase comprised the *uncontrollable* trials where participants experienced a series of uncontrollable aversive tones (4,000 Hz) represented by a different cue compared to the *exposure* trials. The last phase comprised the *controllable* trials where participants were given a series of controllable aversive tones (4,000 Hz) paired with yet another novel cue. **(B)** *Example trial.* In each trial, regardless of the experimental phase, participants were presented a cue (all experimental cues shown for completeness) displayed for 4 s. During the cue presentation for each trial, participants had the option to either press the AVOID or GIVE-UP button. By choosing to press the AVOID button, participants could try to control and avoid the associated tone. A successful AVOID button press yielded no tone presentation whereas a failed AVOID button press yielded 4 s of tone. By choosing the GIVE-UP button, participants would receive a guaranteed 2 s of the associated tone. The cue and tone periods respectively ended with a jittered interstimulus and intertrial interval signaled by a crosshair.

The experiment was divided into four sequential runs each lasting 202 s: two *exposure* phase runs, one *uncontrollable* phase run and one *controllable* phase run (Figure 1A). We had quick verbal check-ins with participants after each run to ensure that they were doing okay in the scanner and was ready to continue onto the next run. We did not explicitly inform participants of the structure of the task (e.g., sequence and nature of the different phases) other than the trial structure. Specifically, all four runs featured identical instructions with no explicit mentioning of controllability or uncontrollability. Each phase also featured a novel cue (i.e., colored shape)—that participants were aware of—paired with either an aversive or a neutral tone (see section “Trial Structure” for further details), which make each phase a novel environment to probe for changes in participants’ avoidance behavior across the runs.

##### Exposure Phase

The exposure phase runs each featured one block of 8 aversive trials and one block of 8 neutral trials. The block presentation order was counterbalanced across participants. All trials in the exposure phase were uncontrollable where participants never received correct feedback (no tone) for any AVOID button presses made in the cue presentation period despite being instructed that they can try to avoid the tone with the AVOID button. Participants received negative feedback (4s tone) to signal that they had no effective behavioral control over the tone presentation. All participants underwent two consecutive exposure phase runs (i.e., early and late runs) with the same set of aversive and neutral cue-tone pairings.

##### Uncontrollable and Controllable Phases

The uncontrollable and controllable phases each comprised 16 aversive trials and introduced new cues that were different from those that the participants experienced in the exposure phase. The uncontrollable phase featured uncontrollable aversive trials where participants always received the incorrect feedback (i.e., 4s tone) for AVOID button presses but never the correct feedback (0s tone). In contrast, the controllable phase provided participants with correct feedback with a deterministic feedback schedule (that was not revealed to participants and does not depend on the timing of AVOID button press)—the first AVOID button press always yielded an incorrect feedback and each subsequent AVOID button press was on an interleaved 50% correct/incorrect schedule. We set the outcome for the first AVOID press to be incorrect to account for participants making an AVOID press by accident. In addition, we chose this schedule to ensure that all participants received the same order of positive and negative feedback regardless of which trial number they made an initial AVOID button press.

#### Trial Structure

A trial (Figure 1B) consisted of a 4s cue period and a 4s tone period, separated by a jittered 1–5 s inter-stimulus interval (ISI) and concluded with a jittered 1–5 s inter-trial interval (ITI). All trial structures were consistent across experimental phases.

Within the cue period, participants were shown one of four different visual cues represented by shapes of different color (e.g., green square). These cues were individually paired with either the aversive or neutral tone. During cue presentation, participants were instructed to make a choice between pressing the AVOID or the GIVE-UP button. The AVOID button was described to participants as having a correct response for each cue—they had to figure out which specific 1 s interval was the correct timing to press the AVOID button in order to successfully avoid the tone. In reality, the feedback for their AVOID button presses was deterministic so as to permit us to manipulate whether the cue represented a controllable or uncontrollable context. For instance, in an uncontrollable context, participants were never given correct feedback (no tone) for any AVOID button presses, hence inducing them to perceive no behavioral control over the tone outcome.

In comparison, the GIVE-UP button did not have an associated correct/incorrect feedback. Instead, participants were informed that pressing it anytime during the cue period would always lead to the expected presentation of an abbreviated 2s-tone. This abbreviated tone associated with the GIVE-UP button was designed to best mimic animal studies where rodents, in response to uncontrollable contexts, chose the lesser of two evils by becoming behaviorally passive (i.e., no longer exerting effort) and conserving energy to accept the impending aversive outcome. This parallels our task where the act of pressing the GIVE-UP button translates into participants knowingly accept that they would get a shortened but guaranteed tone with no chance of avoiding it.

Participants were instructed that only one button press (the first one) was allowed to register for each cue period. To disincentivize participants from choosing the GIVE-UP button for a shorter experimental duration, 2 s was added to the inter-trial interval (ITI) whenever a GIVE-UP button press was made so that all trials had the same length. Any missed response during the 4s-window cue period was registered as a missed trial that resulted in 4s of tone presentation and carried a $1 monetary penalty on the experimental compensation.

### Neuroimaging Data Acquisition

Images were collected using a 3T Siemens MAGNETOM Trio scanner with the 12-channel head at the Rutgers University Brain Imaging Center (RUBIC). High-resolution structural images encompassing the whole brain were acquired using a T1-weighted magnetization-prepared rapid gradient echo (MPRAGE) sequence (repetition time (TR): 1,900 ms; echo time (TE): 2.52 ms; matrix 256 × 256; field of view (FOV): 256 mm; voxel size 1.0 × 1.0 × 1.0 mm; 176 slices; flip angle: 9°). The blood-oxygenation-level-dependent (BOLD) functional images were obtained using a single-shot T_2_^∗^-weighted echo-planar imaging (EPI) sequence (TR: 2,000 ms; TE: 25 ms; matrix 64 × 64; FOV: 192 mm; voxel size 3.0 × 3.0 × 3.0 mm; 35 slices (0% gap); flip angle: 90°). In addition, B_0_ field maps (TR: 400 ms; TE_1_: 5.19 ms; TE_2_: 7.65 ms; matrix 64 × 64; FOV: 192 mm; voxel size 3.0 × 3.0 × 3.0 mm; 35 slices (0% gap); flip angle: 60°) were collected prior to the functional images to correct for geometric distortion in the functional images.

### FMRI Preprocessing

The neuroimaging data were preprocessed using SPM12 (Ashburner, 2012)^1^. First, we defined the origin of each image to align with the anterior and posterior commissure plane (Ardekani and Bachman, 2009). After we motion-corrected each time series to its first volume, we then performed spatial unwarping to minimize geometric distortions due to susceptibility artifacts (Andersson et al., 2001; Hutton et al., 2002). Next, we coregistered the mean functional image to the anatomical scan and normalized the anatomical using the unified segmentation model (Ashburner and Friston, 2005). The normalized anatomical was subsequently used to reslice the functional data to standard stereotaxic space defined by the Montreal Neurological Institute (MNI). We applied a spatial smoothing at full-width half-maximize of 6 mm to the normalized functional data.

To minimize the impact of head motion on the neuroimaging data, we applied additional preprocessing steps using tools from FSL (FMRIB Software Library version 5.0.4; Smith et al., 2004)^2^. We detected motion spikes using the FSL tools *fsl_motion_outliers*. The motion spikes were evaluated with two metrics: (1) root-mean-square (RMS) intensity difference of each volume relative to the reference volume obtained from the first time point; and (2) frame-wise displacements calculated as the mean RMS change in rotation/translation parameters relative to the same reference volume. We subjected the metric values within a run to a boxplot threshold (75th percentile plus 1.5 times the interquartile range) and labeled volumes as spikes, which were subsequently removed via regression (Satterthwaite et al., 2013; Power et al., 2015). Across all participants, this method removed 6.2% of volumes (range: 0.99–13.6%). After the removal of motion spikes, no participants exhibited extreme average volume-to-volume head motion (M = 0.058 mm; range: 0.027–0.10 mm) or maximum volume-to-volume head motion (M = 0.13mm; range: 0.060–0.26 mm). Following the removal of motion spikes, we extracted brain material from the functional images (Smith, 2002) and normalized the entire 4D dataset using a single scaling factor (grand-mean intensity scaling). Images were then processed through the SUSAN (Smallest Univalue Segment Assimilating Nucleus) noise reduction filter, part of the FSL software package, using a 2 mm kernel (Smith and Brady, 1997). This step allowed us to achieve greater signal-to-noise ratio while preserving the image structure. Lastly, we applied a high-pass temporal filter with a 100 s cutoff (Gaussian-weighted least-squares straight line fitting, with sigma = 50 s) to remove low frequency drift in the MR signal. Applying the temporal filter after the removal of motion spikes helps to minimize ringing artifacts (Weissenbacher et al., 2009; Carp, 2013; Satterthwaite et al., 2013).

### Data Analysis

#### Behavioral Analysis of Choices in the CAD Task

We were primarily interested in participants’ avoidance behavior toward the aversive tone across the different experimental phases (i.e., *exposure, uncontrollable, and controllable*). First, in the *exposure* phase, we tested for differences in both total avoidance attempts and changes in avoidance behavior over time associated with the aversive and neutral tones. Second, using a novel cue in the *uncontrollable* phase, we studied the development of passivity and their reduced avoidance motivation in participants exposed to aversive contexts. And finally, by presenting participants with a controllable but aversive context in the last *controllable* phase, we investigated the protective effects of controllability on avoidance behavior.

##### Exposure Phase

We first established any potential differences in participants subjective tone ratings and their AVOID button presses between the aversive and neutral trials using paired t-tests. We then conducted a repeated-measure two-way ANOVA examining the effect of time (i.e., early vs. late run) and cue type (i.e., aversive vs. neutral) on the number of AVOID button presses. Overall, we hypothesized that participants would rate the 4,000 Hz tone to be more aversive and show more avoidance behavior in the aversive compared to the neutral trials, particularly in the early aversive trials.

##### Uncontrollable and Controllable Phases

We first compared the aversive trials in the *exposure* and *uncontrollable* phases utilizing a paired *t*-test to examine changes in avoidance behavior. We hypothesized that participants would show more avoidance behavior in the *exposure* compared to the *uncontrollable* phase. Furthermore, we modeled participants’ proportion of AVOID button presses in the *exposure* phase into a probit regression to investigate whether the aversive or neutral *exposure* avoidance behavior predicted participants’ avoidance behavior in the *uncontrollable* phase. We hypothesized that the aversive *exposure* compared to the neutral trials would better predict participants’ avoidance behavior in the *uncontrollable* phase.

For the *controllable* phase, we implemented a paired *t*-test to probe any differences in the proportion of AVOID button presses made in the *uncontrollable* compared to *controllable* phase. We predicted that participants would demonstrate more avoidance behavior in the *controllable* compared to the *uncontrollable* trials. Finally, to examine the avoidance behavior across all three aversive phases, we analyzed the proportion of AVOID button presses using time as the factor (i.e., early *exposure*, late *exposure*, *uncontrollable, controllable*) in a repeated-measure one-way ANOVA model to test for behavioral differences.

#### Neuroimaging Analysis

Neuroimaging analyses were carried out with FSL FEAT (FMRI Expert Analysis Tool) Version 6.0 (Smith et al., 2004). All of the general linear models (GLM) described below included a reaction time (RT) regressor of no-interest for the cue period with the duration set to the RT of a button press in each cue period and an intensity of one. We regressed out the RTs for each cue period in order to remove RT-related confounds that were unrelated to participants’ choices between AVOID and GIVE-UP presses. All GLMs described below also included regressors of no-interest for any missed trials for the cue period with the duration set to 4 s and an intensity of one. For the first-level analysis, the regressors in all the general linear models (GLM) were convolved with the canonical hemodynamic response function and incorporated temporal derivatives and temporal filtering.

At the group-level analysis, we performed a mixed-effects one-sample *t*-tests using FEAT’s FLAME 1 + 2, which first fits the model using Bayesian modeling for mixed-effects variance estimation before processing all voxels that were close to threshold using the Metropolis-Hastings Markov Chain Monte Carlo sampling to obtain a more precise estimation of the mixed-effect variance (Woolrich et al., 2004). Unless stated otherwise, for all *z*-statistics images discussed, we thresholded and corrected for multiple comparisons across the whole brain using a false-discovery rate-corrected voxel-extent threshold of *p* < 0.05 (Worsley, 2001; Lieberman and Cunningham, 2009). We used MRIcroN and MRIcroGL to create the statistical overlay images (Rorden et al., 2007)^3^.

##### Exposure Phase

For the *exposure* phase, we were interested in differences in neural responses toward aversive and neutral cues in the early and late trials. To investigate this question, we performed a 2 (aversive vs. neutral) × 2 (early vs. late) ANOVA. This ANOVA allowed us to probe whether the context (i.e., aversive or neutral) influenced participants to exhibit different cue responses to the initial and latter stages of learning to avoid uncontrollable tones. Building on our behavioral predictions, we hypothesized that participants would react to the aversive cue more unfavorably, particularly in the late trials when they have learned that the cue was unavoidable, due to the 2-fold setbacks of aversive context coupled with uncontrollability. Based on previous studies implicating the amygdala and ventral striatum in aversive learning (Schoenbaum and Setlow, 2003; Kienast et al., 2008), we hypothesized that participants would show greater activation in these regions when we examine the contrast of aversive – neutral × late – early interaction.

For the first-level GLM analysis, we modeled participant-specific design matrices for each run with the following regressors: (1) a linear regressor encoding the aversive cue period with duration corresponding to 4 s and intensity set to one; (2) a linear regressor encoding the neutral cue period with duration corresponding to 4 s and intensity set to one; (3) a linear regressor encoding the aversive tone period with duration corresponding to 4 s and intensity set to one; (4) a linear regressor encoding the neutral tone period with duration corresponding to 4 s and intensity set to one. This model also included RT regressors of no-interest with duration set to the RT for the button press during the cue period and intensity of one. In addition, we also added nuisance regressors for any missed trials occurring in the cue and tone periods with the duration set to 4 s and an intensity of one. For the first-level model, we created the following contrasts: (1) aversive – neutral cue period; (2) aversive – neutral tone period; (3) aversive + neutral cue period; (4) aversive + neutral tone period. Accordingly, the first-level contrasts allowed us to model the aversive and neutral trials separately.

In the second-level analysis, we used a fixed-effects model to either combine the data across the two runs or contrasted the early and late trials. This setup resulted in three second-level contrasts: (1) early + late; (2) early – late; (3) late – early. In effect, the second level contrasts permitted us to model the temporal element of the task.

In the group-level analysis, we added a participant-specific covariate corresponding to their subjective rating difference between the aversive and neutral tones. This covariate was included to account for the subjective differences in tone perception. We performed a mixed-effects ANOVA to test the main effects of time (late – early) and cue type (aversive – neutral) as well as the interaction between these two factors.

##### Uncontrollable and Controllable Phases

For the *uncontrollable* and *controllable* phases, we wanted to examine differential neural responses toward uncontrollable and controllable cues in the *uncontrollable* and *controllable* phases respectively. We hypothesized that in the *uncontrollable*—*controllable* contrast, participants would recruit amygdala and insula. This hypothesis was grounded on previous work suggesting that the loss or lack of perceived control in an aversive context (e.g., receiving painful stimuli) is associated with increased activity in regions related to negative emotion arousal and the anticipation of aversive events (Salomons et al., 2004; Tanaka et al., 2006; Mohr et al., 2008; Alvarez et al., 2015; Bräscher et al., 2016). In addition, based on our prediction that participants would develop passivity in the *uncontrollable* phase, previous animal research has suggested that regions such as the dorsal striatum and amygdala might be involved in the neural mechanism subserving passivity (Thierry et al., 1976; Maier et al., 1993; Strong et al., 2011; Clark et al., 2014). On the other hand, in the *controllable*—*uncontrollable* contrast, we anticipated that participants would have greater activity in the ventral striatum (i.e., nucleus accumbens) and vmPFC. This prediction was based on our previous finding (Wang and Delgado, 2019) and others (e.g., Maier and Watkins, 2010; Leotti and Delgado, 2011) showing that the nucleus accumbens and vmPFC served as key nodes in the neural circuitry for perceived control. To test these hypotheses, we performed a GLM contrasting the *uncontrollable* and *controllable* trials.

For the first-level analysis, we modeled participant-specific design matrices with the following regressors: (1) a linear regressor encoding the cue period with duration corresponding to 4 s and intensity set to one; (2) a linear regressor encoding the tone period with duration corresponding to 4 s and intensity set to one. This model also included RT regressors of no-interest with duration set to the RT for the button press in the cue period and intensity of one as well as nuisance regressors for any missed trials occurring in the cue and tone periods with the duration set to 4 s and an intensity of one.

In the second-level analysis, using a fixed-effects model, we compared the two experimental phases by creating two contrasts: (1) *uncontrollable*—*controllable*; (2) *controllable*—*uncontrollable*. Finally, in the group-level analysis, we added a participant-specific covariate accounting for their subjective tone rating for the aversive tone. We carried out mixed-effects *t*-tests to examine differences in neural activation in cue period between the two experimental conditions (i.e., *controllable* and *uncontrollable* phases). In addition, we also applied a vmPFC ROI based on our previous finding (Wang and Delgado, 2019) to examine whether vmPFC activity exhibited a significant relationship with participants’ changes in avoidance behavior between the *controllable* and *uncontrollable* phases.

## Results

### Behavioral Results

Prior to the experiment, participants rated the aversiveness of each tone on a Likert scale of 1–7. Participants on average rated the aversive tone (M = 5.55, *SD* = 1.41) significantly higher than the neutral tone (M = 2.09, *SD* = 1.02); [*t*(21) = 9.80, *p* < 0.0001, Cohen’s *d* = 2.08].

#### Aversive vs. Neutral Exposure Trials

**FIGURE 2:**
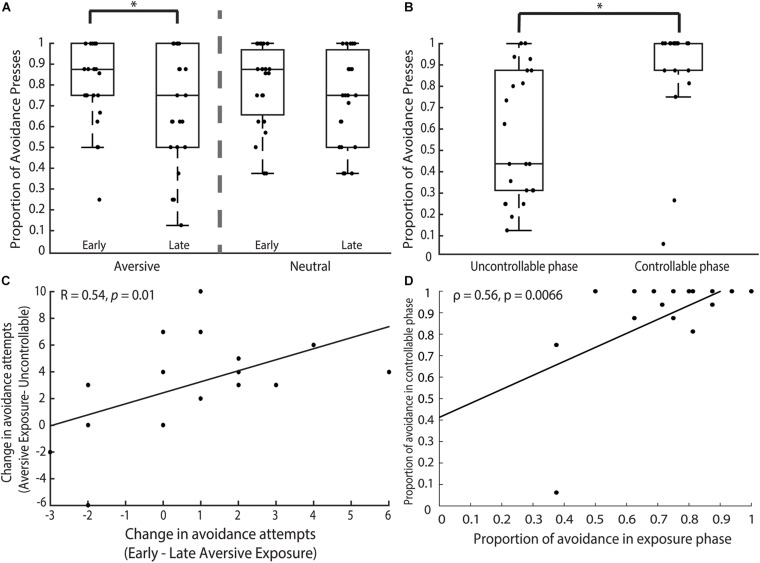
Behavioral findings. **(A)** *Exposure phase.* Participants’ avoidance behavior revealed a significant main effect of time. Pairwise comparisons showed that this significant effect was driven by the marked decrease in AVOID button presses unilaterally present in the aversive but not neutral trials. **(B)** *Uncontrollable and controllable phases*. Participants significantly increased their avoidance behavior in the *controllable* phase when compared to the *uncontrollable* phase. **(C)** We examined participants’ changes in avoidance behavior between the early and late aversive *exposure* trials compared to the same changes in behavior across *exposure* (combined trials) and *uncontrollable* trials. We found that participants who showed greater decrease in their avoidance behavior from the early to late aversive *exposure* trials (i.e., larger x-axis) also showed greater decrease in their avoidance behavior from the aversive *exposure* to *uncontrollable* trials (i.e., larger y-axis). **(D)** We investigated the relationship between the proportion of avoidance behavior in the *exposure* and *controllable* phases and found that participants who made more avoidance presses in the *exposure* phase also subsequently made more avoidance presses in the *controllable* phase. Result remained significant even after removing outllier. *p < 0.05.

In the *exposure* phase, participants made more AVOID than GIVE-UP presses during both the aversive (AVOID: M = 12.09, *SD* = 2.94; GIVE-UP: M = 3.64, *SD* = 3.05) and neutral (AVOID: M = 12.09, *SD* = 2.79; GIVE-UP: M = 3.64, *SD* = 2.84) trials, without any difference in AVOID button presses between the two trial types [*t*(21) = 0.00, *p* = 1.00]. We ran a 2 × 2 ANOVA to examine the effects of trial type (aversive vs. neutral) and time (early vs. late) on the proportion of AVOID button presses. We did not find a significant interaction between trial type and time [*F*(1, 66) = 0.19, *p* = 0.67] or a main effect of trial type [*F*(1, 66) = 0.02, *p* = 0.89]. However, there was a significant main effect of time [Figure 2A; *F*(1, 66) = 6.31, *p* = 0.014] and *post hoc* pairwise comparisons of mean revealed a difference between the early and late aversive trials (*p* = 0.041, Cohen’s *d* = 2.04) but not the neutral trials (*p* = 0.15) where participants made more avoidance responses in the early trials.

#### Uncontrollable vs. Controllable Phases

In the *uncontrollable* phase, participants made more AVOID (M = 9.14, *SD* = 4.75) than GIVE-UP presses (M = 6.59, *SD* = 4.84) in a novel aversive context. In the *controllable* phase, the participants similarly made more AVOID (M = 14.64, *SD* = 3.26) than GIVE-UP presses (M = 1.27, *SD* = 3.28). Using a non-parametric Mann-Whitney test, we found that participants had a higher proportion of AVOID button presses in the *controllable* compared to the *uncontrollable* phase (Figure 2B; *z* = 4.17, *p* < 0.0001), alluding to increases in avoidance behavior that was driven by the presence of controllability in an otherwise aversive but novel context. Note that two participants could be classified as outliers based on the criterion of less than 3 *SD* from mean, but importantly, removing them from analysis does not affect the reported results.

#### Observations Across Time

Examining the avoidance behavior across the three experimental phases (i.e., early and late *exposure, uncontrollable, controllable*), we used a Kruskal-Wallis rank test and found a significant effect of time [X ^2^(3) = 21.2, *p* = 0.0001]. We conducted *post hoc* pairwise comparisons using Dunn’s test (Dunn, 1964) and found a significant difference between early *exposure* and *uncontrollable* (*z* = 2.38, *p* = 0.0086) as well as late *exposure* and *controllable* (*z* = −2.96, *p* = 0.0016) phases. We also observed significant difference between early *exposure* and *controllable* (*z* = −2.13, *p* = 0.017) and a marginally significant difference between late *exposure* and *uncontrollable* (*z* = 1.55, *p* = 0.06) phases.

Looking specifically at the aversive exposure and uncontrollable phases, we found that participants made significantly fewer proportion of AVOID button presses in the *uncontrollable* compared to the *exposure* trials (*z* = −1.99, *p* = 0.047), suggesting that participants showed the development of passivity marked by less avoidance and more giving-up behavior in response to uncontrollable aversive cues over time. Interestingly, we also found that the total proportion of AVOID button presses during the *exposure* phase predicted avoidance behavior in the *uncontrollable* phase, but only for aversive *exposure* trials (β = 2.43; z = 3.96; *p* < 0.0001) but not neutral trials (β = 0.79; z = 1.22; *p* = 0.22). To investigate further, we examined changes in avoidance behavior during aversive trials in the *exposure* and *uncontrollable* phases. Strikingly, we found that participants’ change in avoidance attempts from the early to late aversive *exposure* trials, but not the neutral trials (*r* = 0.33, *p* = 0.14), showed a strong positive relationship with their change in avoidance attempts from the aversive *exposure* to *uncontrollable* trials (Figure 2C; *r* = 0.54, *p* = 0.01), suggesting that those with greater decrease in avoidance behavior between the early and late aversive *exposure* trials also showed greater decrease in avoidance behavior in the *uncontrollable* compared to *exposure* trials. That is, an initial decrease in avoidance predicted increased passivity as the task progressed.

In addition, we investigated the relationship between participants’ avoidance behavior in the *exposure* and *controllable* phases. We found that a significant relationship (Figure 2D; ρ = 0.56, *p* = 0.0066)—that remained significant even after removing the outlier (ρ = 0.48, *p* = 0.026)—where individuals who made more avoidance behavior in the exposure phase also made more avoidance behavior in the subsequent controllable phase. This suggested a negative relationship between passivity and controllability where individuals who were less passive during the exposure phase were more likely to make avoidance behavior in the *controllable* phase, supporting the overarching notion that early passive behavior in the experiment was related to less protection from controllability on avoidance motivation.

These results collectively depicted a behavioral pattern where participants showed a significant decrease in avoidance behavior from the early *exposure* to *uncontrollable* trials, all of which were uncontrollable but novel aversive contexts. However, participants increased their avoidance behavior in the *controllable* phase when controllability was present in a novel aversive context and their avoidance behavior was on par with what they showed in the early *exposure* trials, suggesting that controllability served protective effects to rescue participants’ avoidance behavior.

### Neuroimaging Results

#### Exposure Phase

In the *exposure* phase, we were interested in neural activation due to a sustained aversive context. In our 2 (aversive vs. neutral) × 2 (early vs. late) ANOVA, we did not find any regions that survived multiple comparisons for the interaction or main effects of trial type and time.

#### Uncontrollable and Controllable Phases

**FIGURE 3:**
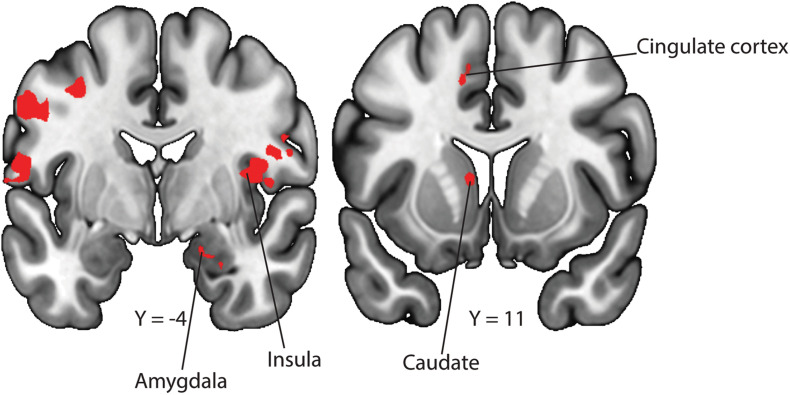
Neural correlates for uncontrollable cues. To examine differences in neural activation between the *uncontrollable* and *controllable* phases, we conducted a GLM contrasting the uncontrollable—controllable trials. We observed activity in the amygdala, insula, cingulate cortex and caudate after correcting for multiple comparisons.

In our behavioral findings, we showed that participants made significantly more avoidance behaviors in the *controllable* compared to the *uncontrollable* phase, suggesting that participants recognized the difference in controllability between the two contexts. We performed a GLM to examine whether there exist differences in neural activation toward the *controllable* and *uncontrollable* cues. In the contrast of *uncontrollable*—*controllable*, we found neural activation in the amygdala (Figure 3; peak *z*-stats = 3.4 at MNI_x, y, z_ = 19, −4, −20, *p*_FDR voxel–corrected_ < 0.05), insula (Figure 3; peak *z*-stats = 4.0 at MNI_x, y, z_ = 42, 0, 7, *p*_FDR voxel–corrected_ < 0.05), cingulate cortex (Figure 3; peak *z*-stats = 4.6 at MNI_x, y, z_ = -12, 12, 44, *p*_FDR voxel–corrected_ < 0.05) and caudate nucleus (Figure 3; peak *z*-stats = 5.0 at MNI_x, y, z_ = −10, 11, 7, *p*_FDR voxel–corrected_ < 0.05). On the other hand, in the contrast of *controllable*—*uncontrollable*, we did not find any region that survived correction for multiple comparisons.

**FIGURE 4:**
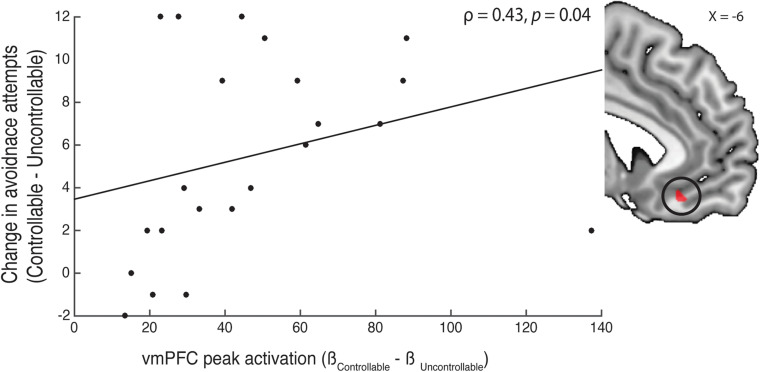
Correlation of avoidance behavior and neural activity. We examined the relationship between vmPFC activity in the *controllable*—*uncontrollable* cue contrast and participants’ changes in avoidance behavior between the two phases. Using a functional mask (shown in the right insert) created from the peak coordinate (peak MNI_x, y, z_ = −6, 32, −14) reported in Wang and Delgado (2019), we extracted the peak activation and correlated them with participants’ change in avoidance attempts from the *controllable* to *uncontrollable* phase. We found that participants with a larger vmPFC peak activation also had a bigger increase in avoidance behavior in the *controllable* trials.

As we did not observe significant activation in the contrast of *controllable*—*uncontrollable*, we conducted an exploratory follow-up analysis with a specific vmPFC ROI. In a previous study (Wang and Delgado, 2019), we reported that the subjective value of control in the appetitive domain was tracked in the vmPFC. Given that our current experiment investigated how controllability influenced avoidance behavior, we were interested in examining whether vmPFC activity in the controllable context showed any relationship with participants’ avoidance behavior. Specifically, in the contrast between *controllable* and *uncontrollable* cues, we used the peak vmPFC coordinate reported by Wang and Delgado (2019; MNIx, y, z = −6, 32, −14) and created a 3 mm region-of-interest functional mask. With this vmPFC mask, we extracted the peak activation (M = 47.07, bootstrap bias-corrected and accelerated 95% confidence interval = [36.80, 61.46]) and correlated this activation with participants’ change in avoidance behavior between the *controllable*—*uncontrollable* trials using a spearman’s correlation (Rousselet and Pernet, 2012). We found that participants who had higher vmPFC peak activation to *controllable* cues also had greater increase in avoidance behavior in the *controllable* compared to *uncontrollable* trials (Figure 4; ρ = 0.43, *p* = 0.04). To investigate whether vmPFC activity in the *controllable* phase can predict participants’ behavior change, we also performed a robust regression to reveal an association between vmPFC activity and behavioral changes (*t* = 4.45, *p* < 0.0001). These findings suggest that participants with stronger vmPFC activation to controllable cues had a correspondingly larger increase in avoidance behavior when presented with a controllable compared to an uncontrollable context.

## Discussion

We investigated the influence of perceived control on rescuing avoidance behavior in aversive contexts and probed the neural basis underlying this behavioral change. We found that after repeated exposure to uncontrollability, participants developed passivity as demonstrated by their decreased avoidance behavior toward a novel aversive cue that was subserved by neural activation in the insula, amygdala and caudate nucleus. However, the presence of control in a subsequent novel aversive context was able to rescue participants’ avoidance behavior and reverse passivity. Importantly, greater change in avoidance behavior in the controllable context was related to stronger activity in the vmPFC.

In the *exposure* phase, we specifically tested for behavioral and neural differences in response to aversive and neutral cues that were both uncontrollable. While participants rated the aversive tone as significantly more aversive compared to the neutral tone, we did not correspondingly find a significant behavioral or neural main effect of cue aversiveness. However, we did find a behavioral main effect of time (early vs. late) that was driven by reduced avoidance behavior in the late compared to early aversive *exposure* trials. Given that our experimental design deviates from prior work using aversive tones (e.g., Büchel et al., 1998), we reason that the uncontrollability nature of the exposure trials rendered both cues (associated with either aversive or neutral tones) to be perceived as aversive. Indeed, previous research have reported that uncontrollability was regarded as both aversive and undesirable (Rodin, 1986; Grillon et al., 2008; Kim et al., 2017) and served in and of itself as an aversive stimulus to trigger cortisol release (Weiner, 1992; Peters et al., 1998). As such, in our CAD task, participants might have regarded both types of cues as equally aversive over time due to their inherent uncontrollability. A potential way to start probing this presumption would have been to ask participants to subjectively rate the aversiveness of the two cues at the conclusion of each *exposure* experimental run. Their subjective ratings would have offered some insights into participants’ subjective perception of the two types of cues.

During the *exposure* phase, we reported a significant decrease in avoidance behavior that was unilaterally present in the aversive but not the neutral trials. Coupled to this finding was the observation that participants made significantly fewer avoidance attempts in the ensuing *uncontrollable* compared to the *exposure* aversive trials. Together, these results suggest that participants’ avoidance behavior reduced over time as they endured persistent uncontrollability across different aversive contexts. Importantly, this reduction in avoidance behavior was observed in participants’ responses to novel cues (in the *uncontrollable* phase), suggesting that they were in some state of passivity where they showed reduced avoidance motivation and behavior (Maier and Seligman, 2016), in line with previous studies examining learned helplessness in both rodents (Seligman et al., 1975; Anisman and Merali, 2001), dogs (Seligman et al., 1979) and humans (Hiroto, 1974; Hiroto and Seligman, 1975).

Our current experimental design allowed us to show that participants’ behavioral change from the early to late aversive *exposure* trials significantly predicted their subsequent behavioral changes from the aversive *exposure* to *uncontrollable* phase. Notably, only the behavioral change in aversive, rather than the neutral *exposure* trials, predicted participants’ ensuing behavioral change in the *uncontrollable* phase. This finding suggests that participants’ behavioral responses toward aversive cues in the late stages of experiencing uncontrollability mimicked their behavior in the early stages where those who gave up quicker also gave up more over time. That is, those whose avoidance motivation sagged early on in the face of uncontrollable aversiveness were also those whose avoidance motivation showed greater decline in latter stages, potentially hinting at the importance of understanding avoidance motivation in the initial/early stages of experiencing an uncontrollable aversive context. Future studies should aim to replicate and extend our current findings to investigate possible explanations for this observation. We hypothesize that this finding could be partially reconciled if participants who gave up quicker were also less persistent in general, hence alluding to a potential interaction of susceptibility to passivity and the behavioral trait of persistence (Cloninger et al., 1998; Duckworth et al., 2007; Bhanji and Delgado, 2014; Lucas et al., 2015).

In addition to the aforementioned behavioral findings, we also observed that the *uncontrollable* phase elicited activation in neural regions such as the insula, amygdala, cingulate cortex and caudate nucleus. This is in line with prior work showing that uncontrollability is undesirable and induces negative emotions (Chorpita and Barlow, 1998; Robbins, 2005; Sanjuán and Magallares, 2009; Pryce et al., 2012). Indeed, uncontrollability can be aversive in and of itself to drive activity in neural regions subserving aversive processing (Hayes et al., 2014). Moreover, the amygdala and caudate nucleus observations are consistent with prior animal studies reporting that these regions contribute to the neural circuitry underlying the behavioral consequences of uncontrollable aversive stimulus, particularly the role of serotonergic activity within these regions in mediating the learning deficits induced by uncontrollability (Amat et al., 1998; Strong et al., 2011). Furthermore, our current findings also support the notion that these regions serve important roles in avoidance learning (Delgado et al., 2009; Choi et al., 2010; Palminteri et al., 2012; Lewis et al., 2013; Atlas et al., 2016).

Across the course of the experiment, participants showed progressively fewer avoidance behaviors, reaching the lowest proportion of avoidance behavior in the *uncontrollable* phase before significantly rebounding in the *controllable* phase. This suggests that presence of control was potent enough to reverse passivity and rescue avoidance behavior even after exposure to persistent uncontrollability in an aversive domain. However, we note that there was one participant whose behavior did not rebound in the *controllable* phase. Instead, this participant exhibited the classic learned helplessness behavior where they demonstrated passivity even when control was present. Although this participant was a behavioral outlier in our current study, their behavior points to the individual differences that exist where presence of control does not exert the same protective effects for everyone across the board. This is an interesting question for a future study with a larger sample to probe personality traits and behavioral tendencies that could make an individual more resistant to the protective effects of control.

In the *controllable* phase, participants who rebounded the most in terms of avoidance behavior were the ones who responded the strongest to the protective effects of controllability. We inferred that these individuals have a stronger desire for control and would accordingly tend to seek out control in the environment, perhaps driven by a stronger coping and resilient tendency (Maier and Watkins, 2010). However, we did not, as hypothesized, observe significant activation in the ventral striatum and vmPFC when in the contrast of *controllable*—*uncontrollable*. As we did not observe significant activation, in our follow-up exploratory analysis with a functional vmPFC ROI from a previous study (Wang and Delgado, 2019), we considered differences in participants’ avoidance between the *uncontrollable* and *controllable* phases. Specifically in the *controllable* phase, we found that vmPFC activity, which was shown in our previous work to encode participants’ subjective value of control (Wang and Delgado, 2019), positively predicted participants’ avoidance behavioral change in the *controllable* phase compared to the *uncontrollable* phase. This finding, which would benefit from replication using a larger sample size, supports the hypothesis that a neural region (i.e., vmPFC) associated with encoding perceived control could predict participants’ changes in avoidance behavior when control is present. However, this does not preclude the possibility that, specifically within an aversive domain, perceived control could recruit specific subregions within the vmPFC or cortical areas such as the ACC—a hypothesis that will benefit from future work. In addition, future studies could also consider the possibility that perceived control could alter the functional connectivity between regions that might respond to the opportunity to exert control in an aversive domain.

Regardless, inferring from our current finding, we reason that how much individuals subjectively value control could help to predict the protective effect of control on their avoidance motivation, an effect that could persist—pending future investigations—even if the environment becomes uncontrollable again. Based on prior work on learned helplessness (Maier and Seligman, 2016), it is possible that successful avoidance in the *controllable* phase would increase the amount of persistence to avoid in a subsequent uncontrollable run where individuals who performed more avoidance behavior in the *controllable* run would be more protected in subsequent uncontrollable environments. Appreciating the protective effect of perceived control could have implications on understanding an individual’s vulnerability toward developing psychopathologies associated with control deficits such as depression (Li et al., 2011; Cheng et al., 2013; Vollmayr and Gass, 2013; Romaniuk et al., 2019) and treatment strategies that aim to enhance subjective perception of control (Kadden and Litt, 2011). In conclusion, we found that an enhanced perception of control influences behavioral passivity and encourages avoidance behavior in an aversive context, a potentially protective effect that is subserved by activity in the vmPFC.

## Data Availability Statement

The raw data supporting the conclusions of this article will be made available by the authors, without undue reservation.

## Ethics Statement

The studies involving human participants were reviewed and approved by the Rutgers University Institutional Review Board. The patients/participants provided their written informed consent to participate in this study.

## Author Contributions

KW and MD contributed to conception and design of the study. KW collected the data, performed the statistical analysis, and wrote the first draft of the manuscript. Both authors contributed to manuscript revision, read, and approved the submitted version.

## Conflict of Interest

The authors declare that the research was conducted in the absence of any commercial or financial relationships that could be construed as a potential conflict of interest.

## Footnotes

1 http://www.fil.ion.ucl.ac.uk/spm/software/spm12

2 http://www.fmrib.ox.ac.uk/fsl

3 https://www.mccauslandcenter.sc.edu/crnl/tools
